# Availability and Accuracy of EMS Information about Chronic Health and Medications in Cardiac Arrest

**DOI:** 10.5811/westjem.2017.5.33198

**Published:** 2017-07-14

**Authors:** Alexander Foster, Victor Florea, Carol Fahrenbruch, Jennifer Blackwood, Thomas D. Rea

**Affiliations:** * University of Washington School of Medicine, Department of Medicine, University of Washington, Seattle, Washington; † Public Health-Seattle and King County, Emergency Medical Services Division of Public Health, Seattle, Washington

## Abstract

**Introduction:**

Field information available to emergency medical services (EMS) about a patient’s chronic health conditions or medication therapies could help direct patient care or be used to investigate outcome disparities. However, little is known about the field availability or accuracy of information of chronic health conditions or chronic medication treatments in emergent circumstances, especially when the patient cannot serve as an information resource. We evaluated the prehospital availability and accuracy of specific chronic health conditions and medication treatments among out-of-hospital cardiac arrest (OHCA) patients.

**Methods:**

The investigation was a retrospective cohort study of adult persons suffering ventricular fibrillation OHCA treated by EMS in a large metropolitan county from January 1, 2007, to December 31, 2013. The study was designed to determine the availability and accuracy of EMS ascertainment of selected chronic health conditions and medication treatments. We evaluated chronic health conditions of “any heart disease,” congestive heart failure (CHF), and diabetes and medication treatments of beta blockers and loop diuretics using two distinct sources: 1) EMS report, and 2) hospital record specific to the OHCA event. Because hospital information was considered the gold standard, we restricted the primary analysis to those who were admitted to hospital.

**Results:**

Of the 1,496 initially eligible patients, 387 could not be resuscitated and were pronounced dead in the field, one patient was left alive at scene due to Physician’s Orders for Life-sustaining Treatment (POLST) orders, 125 expired in the emergency department (n=125), and 983 were admitted to hospital. A total of 832 of 1,496 (55.6%) had both sources of data for comparison and comprised the primary analytic group. Using the hospital record as the gold standard, EMS ascertainment had a sensitivity of 0.79 (304/384) and a specificity of 0.88 (218/248) for any prior heart disease; sensitivity 0.45 (47/105) and specificity 0.87 (477/516) for CHF; sensitivity 0.71 (143/201) and specificity 0.98 (416/424) for diabetes; sensitivity 0.70 (118/169) and specificity 0.94 (273/290) for beta blockers; sensitivity 0.70 (62/89) and specificity 0.97 (358/370) for loop diuretics.

**Conclusion:**

In this cohort of OHCA, information about selected chronic health conditions and medication treatments based on EMS ascertainment was available for many patients, generally revealing moderate sensitivity and greater specificity.

## INTRODUCTION

Chronic health conditions and medications can provide important clinical insight when triaging, diagnosing, and treating patients with critical, emergent illness. In life-threatening emergencies however, the patient is often not able to provide information, and clinicians must use other sources of information. Medical records are often available in the hospital setting. However emergency medical services (EMS) providers must gather information from the family, other bystanders, and/or medications containers in an effort to construct a chronic health profile. Cardiac arrest represents an extreme instance where the patient will uniformly be unable to provide clinical history or medication treatments. Thus, the out-of-hospital cardiac arrest (OHCA) circumstance provides an instructive condition to evaluate the availability and accuracy of this information in the pre-hospital setting.

Moreover, resuscitation from OHCA generally follows a singular algorithm that often ignores the heterogeneous acute pathophysiology.^1^ Although there is no clinical evidence that a patient-specific approach can affect outcome, there is increasing experimental evidence that targeting an individual’s specific pathophysiology may result in greater likelihood of successful resuscitation.^2^,^3^ To this end, chronic comorbidity and medication treatment can affect arrest pathophysiology, response to care, and the likelihood of survival.^4^–^8^

Investigation regarding whether and how chronic comorbidity may influence pathophysiology and response to treatment depends in part on the field availability and accuracy of such information. To date, there are no published investigations on the availability or accuracy of field information related to chronic comorbidities and medication treatments in cases of OHCA. The purpose of the present study is to evaluate the availability and accuracy of field information about selected underlying chronic health conditions and medication treatments in patients suffering ventricular fibrillation OHCA.

## METHODS

### Study design, population, and setting

This was a retrospective cohort study of persons 18 years or older who suffered a non-traumatic cardiac arrest and presented to EMS with an initial rhythm of ventricular fibrillation in a large metropolitan EMS system between January 1, 2007, to December 31, 2013 (n=1496). We a priori chose out-of-hospital ventricular fibrillation cardiac arrest because this group provides a well-defined benchmark group that would uniformly be unable to provide a first-person account of chronic health issues or medication treatments.^9^ Because we used the hospital record as the gold standard to determine the presence of chronic health conditions and medication treatments, the primary analyses excluded those who could not be resuscitated and were pronounced dead in the field (n=387), one patient who was left alive at scene due to Physician’s Orders for Life-sustaining Treatment (POLST) orders, and those transported to hospital who died in the emergency department (ED) (n=125). The remaining 983 were admitted to hospital and served as the eligible analysis cohort.

Population Health Research Capsule:

What do we already know about this issue?

Little is known about the availability and accuracy of information collected by EMS in the prehospital setting regarding chronic health conditions or chronic medication treatments.

What was the research question?

The study compared prehospital information with the hospital record to determine agreement for patients suffering out-of-hospital cardiac arrest.

What was the major finding of the study?

EMS information was available for many patients and had moderate sensitivity and greater specificity.

How does this improve population health?

The findings suggest that EMS information about chronic health conditions and medication treatments has ample accuracy to be considered for research or help inform clinical decisions.

The study EMS system is a two-tiered system that serves 1.2 million people in urban, suburban, and rural settings. The first tier of EMS response is firefighter emergency medical technicians equipped with automated external defibrillators. The second tier is paramedics who practice advanced care life support. This study was approved by the appropriate institutional review board.

### Data collection

The EMS maintains a registry of every treated OHCA organized according to the Utstein template.^9^ Information is collected from dispatch, EMS, hospital, and death certificate records. The study was designed in keeping with chart-review research guidelines.^10^ We used a uniform data collection form to ascertain information about chronic health conditions and chronic medication treatment (preceding the OHCA). Information was collected from the EMS record and the hospital records independently. Specifically, we collected information about chronic health conditions and particular classes of medications using a uniform abstraction form (Appendix 1). Abstraction was aided by a written guideline (Appendix 2) that provided generic, trade name, and medication class as well as rules for coding particular chronic health conditions. Abstractors were not aware of the specific hypotheses, which data elements were of primary interest, or how the data elements would be formally tested.

We coded comorbidities as present if noted to be present in the narrative of the prehospital medical incident report form completed by paramedics, or by the notation of a medication used for that specific condition if medication information was available in the absence of any other information. Conditions were deemed absent if they were stated to be absent or if they were not mentioned in the context of a documented medical history. We classified clinical condition or medication as unknown if stated to be “unknown” or if there was no information about medication treatment or chronic health conditions from the record. For hospital data collection, we used the entire hospital record for the OHCA hospitalization to assess comorbidities and medication treatments prior to the OHCA event.

### Data Definitions

We defined heart disease as any prior cardiac comorbidity or cardiac procedure. A history of hypertension or hyperlipidemia was not considered to be heart disease. Further, the determination of any heart disease from the prehospital records included the notation of any cardiac medication such as nitroglycerin or digoxin in the absence of other information about a patient history. As part of the process there was an evaluation of inter-reviewer reliability to assure consistency in the abstraction. At the outset, each abstractor independently reviewed a common set of 20 cases. There was 90% agreement with regard to the diagnosis of any heart disease from the EMS report between the two reviewers. We a priori chose to evaluate chronic health conditions associated with cardiac arrest to include any heart disease, congestive heart failure (CHF), diabetes, beta blocker use, and diuretic use specific to loop diuretics (furosemide – trade name Lasix; bumetanide – trade name Bumex; and torsemide – trade names Demadex, Diuver, and Examide).

### Data analysis

We used the hospital record as the gold standard when identifying medical conditions and medications. We compared independent proportions of categorical variables with the chi-square statistic or Fisher’s exact test, paired categorical comparisons with the McNemar’s test, comparisons of independent continuous variables with the Mann-Whitney nonparametic statistic, and paired comparisons of continuous variables with the paired t-test. We also compared characteristics between patients who died prior to hospitalization and thus lacked hospital information and those with hospital information. We report the characteristics of the 513 who died prior to admission and were excluded from the primary analysis to highlight the differences between those in the primary comparison group and those excluded from the study.

In the primary analyses, we generated 2 × 2 tables to determine sensitivity and specificity of the specific chronic health conditions and medication treatments comparing the EMS information to the hospital information. In generating sensitivity and specificity, we excluded those for whom the information was coded as “missing” or “unknown” in the EMS record. All analyses were conducted with SPSS Version 21.^11^

## RESULTS

Of the 1,496 adult non-traumatic arrests that presented to EMS with an initial shockable rhythm during the study period, 1,360 (91%) had an EMS report available for abstraction. Of these 1,360, 1,129 (83%) had at least some information about chronic health conditions and 885 (78%) had at least some information about medication therapies. Of the 1,496 patients, 1,108 were transported to hospital for continued treatment, and 983 were admitted. Among the eligible 983 admitted patients, 910 (92.6%) had a comorbidity form, and 903 (91.9%) had a hospital record abstraction form; 832 (84.6%) had both sources of data for comparison and comprised the primary analytic group.

Compared to those who survived to hospital admission, those pronounced dead in the field or the ED (so excluded from the primary analyses) were older, more likely to suffer the arrest in a residential setting, less likely to be witnessed or receive bystander cardiopulmonary resuscitation (CPR), and more likely to have comorbidity information reported on their EMS reports. The group who died prior to admission also was more likely to be treated with beta blockers or loop diuretics (Table 1).

For the comparison of clinical comorbidity, approximately 75% (638/832) had known status regarding the three comorbidity conditions from the EMS and hospital information The large majority of missing information was from the EMS record (98%, 191/194). Missing or unknown information for any of the three comorbidity variables from the EMS record was greater among OHCA occurring in public locations (43%, 138/320) compared to residential locations (10%, 53/510). Agreement between EMS and hospital records among the selected comorbid conditions was significant. EMS ascertainment of any prior heart disease had a sensitivity of 0.79 (304/384) and a specificity of 0.88 (218/248) using the hospital record as the gold standard (p<0.001). For CHF, sensitivity was 0.45 (47/105) and a specificity was 0.87 (477/516) (p=0.01); and for diabetes, sensitivity was 0.71 (143/201) and specificity was 0.98 (416/424) (p<0.001).

For the comparison of selected medications, 56% of patients (467/832) had known medication status, with the number and types of medications reported on both the EMS and hospital forms. The average number of medications was 3.4 (SD 3.1) by EMS report compared to 5.0 (SD 4.0) for the hospital form, for a mean difference of 1.6 more medications reported on the hospital form (95% CI 1.3 – 1.9, p < 0.001, paired t-test). As with chronic conditions, there was moderate sensitivity and high specificity with regard to beta blocker and diuretic use (Table 2).

## DISCUSSION

In this cohort of persons suffering ventricular fibrillation arrest, we observed variable amounts of information available and recorded by EMS providers. EMS ascertainment of selected chronic health conditions and medications had modest sensitivity and greater specificity when compared to the hospital record.

The field availability of information about chronic health conditions and medication treatments is not well described. In the current investigation, EMS was able to ascertain some information about chronic health conditions for approximately 83% of patients and information about medications in approximately two thirds of patients, though the availability for a specific condition or medication was less. Thus, there is some opportunity for EMS to glean clinical history in the majority of patients, even when the patient cannot be a resource.

Beyond availability, the accuracy of the information about chronic health conditions and medication therapies is also uncertain. In this current study, with the exception of CHF, we found that the selected medical conditions and medications therapies had a sensitivity of approximately 70% and specificity of 90% or greater. Thus EMS correctly identified about 70% who truly possessed a given characteristic. Conversely, about 5–15% of the time EMS incorrectly identified a given characteristic when it was not present (false positive).

Whether more (accurate) information might be available if this questioning was prioritized as part of training is uncertain. The precise timing of the information was not available based on this review. Information that is available late in the field process may have less relevance for field care though it could direct hospital treatment decisions. Moreover, the study community’s EMS system typically has five or more EMS rescuers on scene of a cardiac arrest so that there is potentially more opportunity for this type of information gathering. These qualifications aside, the results suggest that at least some information about chronic health conditions and medications is available during many field resuscitations.

What are the implications of these results? The approach provides an opportunity to understand whether chronic conditions or medication treatments might help explain the OHCA outcome disparity across systems and among patients.^12^. For example, there was a five-fold variation in survival among ventricular fibrillation cardiac arrest cases across the sites participating in the Resuscitation Outcomes Consortium and a 10-fold variation in overall survival among communities participating in the Cardiac Arrest Registry to Enhance Survival.^13^,^14^ The current Utstein data elements explain less than 25% of system differences in survival for ventricular fibrillation.^15^ Whether some of this disparity might be explained by patient substrate as characterized by chronic conditions or medication treatment requires further investigation. Certainly there are individual and community differences in chronic health conditions and access to medical care that might provide the circumstance for important differences that not only influence the risk of incident OHCA but also influence prognosis.^16^,^17^

Moreover, the findings inform the potential to incorporate chronic health conditions into treatment approaches. Although there is currently no strong evidence that selected conditions or medications are amenable to a specific alteration in resuscitation treatment, the concepts are derived from experimental models and modest evidence from observational human investigations.^2^,^5^ The current results suggest that treatment alterations could be implemented in a manner such that a subset with a particular chronic health condition or medication treatment could be correctly identified, but that a sizable subset with the specific condition or medication treatment would be missed based on a substantial false-negative rate. However, there is no clear consensus about what conditions or treatments should be prioritized and how such information collection could be standardized across systems.^18^ Presumably the choice would depend upon the presentation and circumstances.

## LIMITATIONS

This study has several limitations. The gold standard of hospital ascertainment was only available for those who survived to hospital admission. Those who died prior to hospitalization were different based on Utstein measures such as older age, less bystander CPR, and more residential location. Our finding that this group of older, in-residence arrests had a greater prevalence of chronic conditions and medications suggests that EMS ascertainment for those who died prior to admission is at least consistent with the expectation that comorbidity increases with age.

We also a priori selected chronic health conditions and medications that we believed may be relevant to resuscitation pathophysiology and prognosis. Other conditions or medications may produce different results. Outside of the initial inter-reviewer reliability testing and informal group discussions, there was not a repeat formal evaluation of abstraction performance. Additional effort to evaluate and refine inter-reviewer agreement may have improved precision though the initial inter-reviewer reliability was good.

As noted, the investigation involved a large metropolitan EMS system and the results may not be generalizable to other systems. These limitations should be considered in the context of the study’s strengths. The investigation involved a large sample of well-characterized OHCA events, was motivated by an evolving understanding of the pathophysiology and treatment of OHCA, and presented results for a number of conditions and medication treatments.

## CONCLUSION

In this cohort of adult patients with non-traumatic OHCA presenting with ventricular fibrillation, information about selected chronic health conditions and medication treatments based on EMS ascertainment was available for many patients, generally revealing moderate sensitivity and greater specificity. The findings suggest that information about chronic health conditions or medication treatments could be relevant when investigating outcome disparity and potentially help guide therapy for a subset if a directed and individualized approach ultimately has therapeutic rationale. Future efforts may evaluate approaches to increase both the availability and accuracy of field information involving chronic health conditions and medication treatments.

## Supplementary Information





## Figures and Tables

**Table 1:** Case characteristics by status of patients admitted with non-traumatic cardiac arrest, n = 1,496.

	Admitted		Died prior to hospital		
			
	n	%	n	%	p-value
Number of cases	983		513		
Male	748	76.1%	406	79.1%	
Patient age, mean years (SD)	61.1(14.7)		65.5 (15.7)		< 0.001
Circulatory arrest prior to EMS arrival	873	88.8%	468	91.2%	
Witnessed collapse (for arrest on EMS arrival)	690	79.0%	287	61.3%	< 0.001
Bystander CPR (for arrest prior to EMS arrival)	669	76.6%	328	70.1%	< 0.001
Response location					< 0.001
private residence	577	58.7%	322	62.8%	
public location	373	37.9%	140	27.3%	
Care setting: NH, AFH, GH, AL*	33	3.4%	51	9.9%	
Case has a Comorbidity Form (n=1,360)	910	92.6%	450	87.7%	< 0.001
Comorbidity information reported (n=1,360)					< 0.001
any information reported	747	82.1%	382	84.9%	
none reported, or stated unknown	163	17.9%	68	15.1%	
Any heart disease reported (n=1,059 known)	372	53.5%	209	57.4%	0.014
CHF reported (n=1,051 known)	94	13.6%	73	20.3%	< 0.001
Diabetes reported (n=1,052 known)	169	24.5%	99	27.4%	0.01
Number of medications (n=885 known), mean (SD)	3.5(3.3)		3.9(3.0)		< 0.02
Beta blocker listed (n=885 known)	166	29.0%	100	32.1%	< 0.03
Loop diuretic listed (n=885 known)	92	16.1%	73	23.4%	0.025

*NH*, nursing home; *AFH*, adult family home; *GH*, group home; *AL*, assisted living, *SD*, standard deviation; *CHE*, congestive heart failure.

**Table 2:** Sensitivity and specificity of emergency medical services (EMS) report for medication use.

	Hospital	
	
	Positive	Negative
Beta blocker (n=459)*		
EMS		
Positive	118	17
Negative	51	273
Diuretic (n=459)†		
EMS		
Positive	62	12
Negative	27	358

* sensitivity = 0.70, specificity = 0.94, p < 0.001.

† sensitivity = 0.70, specificity = 0.97, p < 0.03.
