# Supplementary material for: Availability and Accuracy of EMS Information about Chronic Health and Medications in Cardiac Arrest
Source: West J Emerg Med. 2017 Jul 14;18(5):864–9. doi: 10.5811/westjem.2017.5.33198 (PMC5576622; doi:10.5811/westjem.2017.5.33198)
Supplement: Supplementary file 1 [file wjem-18-864-s001.docx]

Appendix 1. Comorbidity Data Form

ID# __ __ __ __ __ __ __ __ PT. NAME __ __ __ __ DATE: ___--___--_____ ARREST# __ __

Only for cases < 9/1/2012

__ **(MED) Medications:**

______________________ ______________________ ______________________

______________________ ______________________ ______________________

______________________ ______________________ ______________________

KEY:

1 = Yes, stated to be present

2 = Stated to be absent or Not Stated for an individual condition when other conditions are stated to be present

3 = Yes (derived from med list)

8 = Nothing Stated (do not use for conditions not stated when others are stated to be present– use “2” for “not stated” in this case)

9 = Stated to be Unknown

__ **(CHIST) Chronic Conditions/History:**

__ **(HD) Heart Disease**

__ (CA) Previous Cardiac Arrest

__ (MI) Previous MI/Heart Attack

__ (CHF) CHF/Cardiomyopathy

__ (CAD) Chest Pain/Angina/CAD

__ (ASCVD) Atherosclerosis/Cardiovascular Disease

__ (AFIB) Atrial Fibrillation/Other Arrhythmias

__ (VALV) Valvular Disease

__ (PROC) Heart Procedures (circle): CABG, ICD, pacemaker, PTCA, stent, angio, valve repl,

other: _____________________________________________ (Added 8/20/2014)

__ (HDU) Heart Disease Unspecified (Added 8/20/2014)

__ **(OMH) Other Medical History**

__ (HTN) Hypertension

__ (HCH) High Cholesterol

__ (DIAB) Diabetes

__ (STRK) Stroke

__ (LIV) Liver Disease

__ (GI) Gastrointestinal Disorders

__ (KIDN) Kidney Disease/Dialysis

__ (CANC) Cancer _________________

__ (MH) Mental Health

__ (LUNG) Lung Disease: COPD, asthma, emphysema, home O_2,_ other, unspecified (added 8/2014)

__ (OCC) Other Chronic Conditions: ____________________________________

__ **(OBSRF) Other Observable Risk Factors**

__ (SMK) Smoking __ (OBS) Obesity

__ (SA) Substance Abuse __ (MDSCR) Midline Chest Scar

__ **(SHIST) Symptoms**: (Acute, within 2 days prior to event)

__ (CHP) Chest pain

__ (AMP) Arm/Shoulder/Neck pain

__ (BCK) Back pain

__ (DYSP) Dyspnea

__ (IND) Indigestion/Abd pain

__ (DIZZ) Dizziness/Syncope

__ (NAUS) Nausea

__ (TDN) Tiredness/Weakness

__ (DIA) Diaphoresis

__ (MAL) general Malaise

__ (OS) Other: _______________________

____________________________________

__ (PNU) Recent pneumonia infection **Physical Activity Level**: __ (1) Sleeping

__ (PHYS1) Recent physician visit (w/in 2d) __ (2) Awake – Not Active/Unk

__ (PHYS2) Recent physician visit (w/in 2w) __ (3) Awake – Active

__ (4) Eating

__ (5) Emotional Stress __ (6) Other

__ (7) Unknown

__ (**MED) Medications. Code as described below:**

1=yes, pt takes medications (even if specific types are unknown), 2=no, stated that pt does not take medications, “None” is written into the medications box, or the “None” checkbox is checked; 8=nothing is mentioned about medications, the medications box is empty, and the “None” checkbox is not checked; 9 = stated that it is unknown if the patient takes medications-do not use for specific meds unknown. If the pt takes medications, enter the number taken of each type below.

__ (NSBB) Non-select BetaBlocker

__ (SBB) Select Beta Blocker

__ (MBB) Mixed Beta Blocker

__ (NCCB) Nondihydro Calcium Channel Blockers

__ (DCCB) Dihydro Calcium Channel Blockers

__ (AI) Ace Inhibitor

___ (ARB) ARBs

__ (AB) Alpha Blockers

__ (BP) Blood pressure medications not otherwise

specified

__ (AAR1) Antiarrhythmics Class 1

__ (AAR2) Antiarrhythmics Class 2

__ (DG) Digoxin

__ (DU) Diuretics

__ (LDU) Loop diuretics

__ (TDU) thiazide diuretics

__ (SPI)aldosterone blockers

__ (ODU) Other diuretic

__ (ST) Statins

__ (LLA) Other lipid lowering agents

__ (ASA) Aspirin

__ (AP) Antiplatelets other than ASA

__ (WARF) Warfarin

__ (GLY) Oral hypoglycemics

__ (INS) Insulin

**NOTES:**

__ (APAP) Acetaminophen

__ (NSD) NSAIDS

__ (AINF) Anti-inflammatories other than NSAIDS

__ (PRED) Prednisone

__ (IMM) Immune suppressive agents other than

prednisone

__ (IBA) Inhaled beta agonists

__ (ATRO) Atrovent Inhalers

__ (INH) Other inhalers

__ (AHIST) Antihistamines

__ (HB) H2 Blockers

__ (PPI) Proton Pump Inhibitors

__ (THY) Thyroid medications

__ (EST) Estrogens and Progesterones

__ (SSRI) Antidepressants – SSRIs

__ (TRI) Tricyclics

__ (PSY) Psych meds other than SSRIs & TCAs

__ (ABIO) Antibiotics

__ (NARC) Narcotics

__ (K) Potassium

__ (SS) Stool Softener

__ (XOI) Xanthine Oxidase Inhibitor

__ (OTH) Other: ______________________

__ (QTP) Known QT prolong agents

___ (UNKMEDS) Unk meds, 9 for qty unknown
