# Supplementary material for: Availability and Accuracy of EMS Information about Chronic Health and Medications in Cardiac Arrest
Source: West J Emerg Med. 2017 Jul 14;18(5):864–9. doi: 10.5811/westjem.2017.5.33198 (PMC5576622; doi:10.5811/westjem.2017.5.33198)
Supplement: Supplementary file 2 [file wjem-18-864-s002.doc]

Appendix 2.

DATA ABSTRACTION: GUIDELINES

**Medications**

*Basics:*

1 = yes, medications are listed on one or more report

2 = no medications (“none,” “no,” “no meds,” “0,” or “Ø” is written in medications box or narrative)

Blank = medications box is left blank or “unknown” or “UNK” is written.

*Further Guidelines:*

1. All medications in the medication box are recorded, doing best to work with illegible handwriting and misspellings. Indecipherable meds are flagged to be reviewed by Dr. Tom Rea. If still indecipherable, the med is crossed out and assumed unknown.
2. Drugs that are repeated with different names (e.g. Lasix and furosemide), whether on the same report or one on each report, are considered one drug, and only one is recorded. The exception to this is when the same drug is listed as different dosage forms (e.g. NTG patch and NTG tablets). In this case, both drug forms are recorded.
3. “BP meds”, “Cardiac meds,” etc., that are not otherwise specified are quoted as such, and MED = 1.
4. Medications listed in the narrative, but not in the medication box are recorded.
5. On conflicting reports, written medications trump “none” or “unknown.” “None” trumps “unknown” with the exception of cases where narrative clearly shows that the medications are unknown.
6. Combination meds (Vicodin) allow for the coding of both drugs (oxycodone, acetaminophen)
7. “Prednisone” includes other oral corticosteroids
8. Inhaled corticosteroids are classified as “other inhalers”
9. Atypical antidepressants (buproprion) are coded as physch meds
10. Drugs that are taken “PRN” or “as needed” are included.

**Chronic Conditions/History**

*Basics for Headline:*

1 = yes, chronic conditions or past medical history (PMH) is mentioned on one or more report.

2 = no, one or more of the reports explicitly states the patient has no PMH or no significant PMH on one or more report.

Blank = Chronic conditions or PMH are not mentioned or explicitly stated as unknown.

*Update for new coding form:*

**1=yes**: if the medical record specifically states that the condition exists

**2=no/not stated**: the medical record either explicitly states that the condition is not met (eg, “no heart history” or, in the mind of the reviewer, the record is complete enough *to judge* that the condition was not met (eg, if numerous meds were listed along with one or two other chronic conditions but no heart history was recorded, a “2” would be indicated)

**3=Yes (derived from med list**): If a condition was not stated in the medical record (hyperlipidemia, eg) but the medication list indicates that they have the condition (a statin). This is used mostly with diabetes and statin meds.

**8=nothing stated**: If the medical record is incomplete and it appears that the EMTs/Medics simply did not request the information. This is often used for the observable risk factors since they are often not indicated one way or the other.

**9=Stated as unknown**: this is used when EMTs/Medics indicate that they inquired about a condition or medications but those around the victim did not know one way or the other. This is often used when the arrest is in a public location and witnesses do not intimately know victim. Again, a judgment call may have to be made when some information is given but in the mind of the reviewer it is not complete enough to give a 1 or a 2.

Other Observable Risk Factors:

This category was created when EMTs/Medics are responding to a call when little is known about the victim. Often these will be coded as an “8” since they are not stated one way or the other in the report.

A note on blanks: The new form is hierarchical in nature. “Chronic Conditions History” is an overall heading encompassing the categories of Heart Disease, Other Medical History, and Other Observable Risk Factors. Each of these four fields should be assigned a number, but the individual entries under each subheading can be left blank. In such a case the number on the subheading is understood to code for each of the particular conditions as well.

Notes:

1: “Obese” can be coded when “obese” or a specific high body weight (eg, 350 lbs) is indicated on the record. The body weight needed is left as a judgement call, but it is awknowledged that if it is noted then it is probably because of obesity.

2: “Distended” when talking about the abdomen in a Physical Exam notation **does not** allow for the coding of “obese.” Neither does “soft.”

*Basics for Each Condition:*

1 = yes, the chronic condition is mentioned on one or more report.

2 = no, one or more of the reports explicitly states the patient does not have the condition.

Blank = the condition is not mentioned or explicitly stated as unknown.

*Further Guidelines:*

1. If the Pt is said to have no PMH, with the exception of one or more conditions, than a 1 is marked next to those conditions the patient is said to have, and a 2 is marked next to all other conditions (except smoking, mental health, and substance abuse, which are all left blank since these conditions are often not included on the report, or are not considered PMH).
2. If the Pt is said to have no PMH on one report, but the other report mentions a condition, then the guideline above is followed.
3. “No pertinent history” = no heart history
4. For cardiac procedures, mark 1 and circle the procedures mentioned. These procedures can be used to assess the cardiac history if not otherwise specified in the report. *(Note: So far, I have only done this for valve repair and replacement.)*
5. Cancer: (see question sheet)
6. Lung Cancer = Cancer (and write down type)
7. Gallbladder surgery ≠ Gastrointestinal Disorder
8. Pancreas removal = Diabetes + Gastrointestinal Disorder
9. Non-recent pneumonia infection, amputations ≠ Chronic condition
10. Sometimes “obesity” is abstracted from the objective portion of the report.
11. Alzheimer’s and Dementia fall under “Other,” not “Mental Health”
12. Medication that is clearly given for one condition (insulin=diabetes, statin=high cholesterol) allow for the coding of that condition even in the absents of any other PMH. Code with a “3” in these instances
13. Patient is said to have diabetes even if diabetes has since resolved
14. Bypass surgery qualifies as a chronic GI disorder
15. “overweight” counts as obesity

**Symptoms**

*Basics for Headline:*

1 = yes, one or more symptoms that occurred within the last two days are mentioned (includes symptoms that started more than 2 days prior to the event, but still continued within 2 days of the event).

2 = no, one or more of the reports explicitly states the patient did not have any symptoms or complaints *within 1 hour* of the event (if the patient was awake) or did not have any complaints the night before (if the patient was sleeping).

Blank = Symptoms are not mentioned, explicitly stated as unknown, or the patient is reported to have had no complaints *more than 1 hour* before the event.

*Basics for Each Symptom:*

1 = yes, the symptom is mentioned on one or more report.

2 = no, one or more of the reports explicitly states the patient does not have the symptom.

Blank = the symptom is not mentioned or explicitly stated as unknown.

*Further Guidelines:*

1. If the Pt is said to have no symptoms, with one or more exceptions, than a 1 is marked next to those symptoms the patient is said to have, and a 2 is marked next to all other conditions.
2. If the Pt is said to have no symptoms on one report, but the other report mentions one, then the guideline above is followed.
3. Dyspnea, coughing, and vomiting (even if with blood) are only considered symptoms if they occur well before the collapse or loss of consciousness, as these symptoms can be confused with agonal respirations. If the timing of these symptoms can not be determined and the narrative offers no clues, then the symptom remains unknown.
4. “No cardiac symptoms” = No chest pain or dyspnea
5. Vomiting = Nausea
6. Heartburn = Indigestion
7. Sometimes “diaphoresis” is abstracted from the objective portion of the report.
8. Recent dialysis does not count as a recent physician visit.

**Pneumonia**

*Basics:*

1 = yes, a “recent pneumonia infection” or a pneumonia infection within (what time frame??) is mentioned.

2 = no, one or more reports explicitly states that the patient did not have a recent pneumonia infection.

Blank = A recent pneumonia infection is unknown because pneumonia is not mentioned, the infection mentioned is greater than (???) ago, or the timeframe of the infection is unclear (e.g. pneumonia is listed under PMHx, without reference to the date).

**Recent Physician Visit**

*Basics:*

1 = yes, a recent physician or hospital visit is mentioned, and occurred within the specified time frame (PHYS1 = 2 days or PHYS2 = 2 weeks).

2 = no, one or more reports state that the patient did not see a physician within the specified time, did not see a physician recently, has never seen a physician, or saw a physician, but not within 2 weeks from the time of the event.

Blank = a physician or hospital visit is not mentioned on any report or stated as unknown.

*Further Guidelines:*

1. If the patient is at a clinic at the time of the event, then PHYS1 = 1.
2. If the patient is en-route to the clinic or scheduled to see a physician later the day of the event, then it is considered unknown whether the patient has seen a physician.
3. If the patient is said to have seen a physician recently, but the time is not specified, then PHYS2 = 1.

**Physical Activity Level**

*Basics:*

1 = the physical activity level the patient is at when the event occurred.

All Blank = the physical activity level is not mentioned or unknown.

*Further Guidelines:*

1. If the patient was found on a bed, couch, or chair by the RP, but not specified as to what the patient was doing, the physical activity level is unknown. (Exception: If the patient is found in bed by the RP, but the time of day is between 23:00 – 06:00 and the circumstances in the narrative point to the patient having been asleep, then Sleeping = 1.)
2. If the patient was found by the BLS/ALS unit on the floor, a bed, a couch, or a chair, but not otherwise specified, the physical activity level is unknown.
3. Sleeping = Pt sleeping at time of event or just before (???)
4. Awake–Inactive = Pt found on floor (maybe only if floor is specified?? – depends on sleeping definition) by RP or patient found outside by RP or BLS/ALS, but activity level not otherwise specified; Pt talking to RP or BLS/ALS at time of event; Pt called 911 themselves; Pt found behind wheel of car or in MVA; Pt watching TV, reading, making dinner, etc.
5. Awake-Active = on the treadmill, at the gym, walking outside, shopping, yardwork, etc. either at the time of the event or shortly before. (e.g. Patient came in from doing yardwork, sat down and collapsed).
6. Eating = Pt eating at the time of the event.
7. Emotional stress = Pt experienced some stressful event at time of collapse or just prior; Pt said to have had very stressful day.
8. Other = Other significant activities not included above (e.g. big night of drinking, etc.)

**Other Notes**

In abstracting the data, the above guidelines are followed to the best of my capabilities. Despite the best intentions to follow these guidelines, however, there are still multiple instances where reports are vague or have conflicting information. In these instances I use my best judgment to assess the validity of the information on a case by case basis.
